# Surface Plasmon Resonance Temperature Sensor Based on Photonic Crystal Fibers Randomly Filled with Silver Nanowires

**DOI:** 10.3390/s140916035

**Published:** 2014-08-29

**Authors:** Nannan Luan, Ran Wang, Wenhua Lv, Ying Lu, Jianquan Yao

**Affiliations:** 1 College of Precision Instrument and Optoelectronics Engineering, Institute of Laser and Optoelectronics, Tianjin University, Tianjin 300072, China; E-Mails: wangran1986@tju.edu.cn (R.W.); lvwh66@163.com (W.L.); luying@tju.edu.cn (Y.L.); jqyao@tju.edu.cn (J.Y.); 2 Key Laboratory of Opto-Electronics Information Technology (Ministry of Education), Tianjin University, Tianjin 300072, China; 3 College of Physics and Optoelectronic Engineering, Weifang University, Weifang, Shandong 261061, China

**Keywords:** temperature sensor, fiber optic sensor, photonic crystal fiber, surface plasmon resonance, silver nanowire, mixture

## Abstract

We propose a temperature sensor design based on surface plasmon resonances (SPRs) supported by filling the holes of a six-hole photonic crystal fiber (PCF) with a silver nanowire. A liquid mixture (ethanol and chloroform) with a large thermo-optic coefficient is filled into the PCF holes as sensing medium. The filled silver nanowires can support resonance peaks and the peak will shift when temperature variations induce changes in the refractive indices of the mixture. By measuring the peak shift, the temperature change can be detected. The resonance peak is extremely sensitive to temperature because the refractive index of the filled mixture is close to that of the PCF material. Our numerical results indicate that a temperature sensitivity as high as 4 nm/K can be achieved and that the most sensitive range of the sensor can be tuned by changing the volume ratios of ethanol and chloroform. Moreover, the maximal sensitivity is relatively stable with random filled nanowires, which will be very convenient for the sensor fabrication.

## 1. Introduction

Photonic crystal fibers (PCFs) [1,2], also called holey fibers (HFs) or microstructured optical fibers (MOFs), are composed of a periodic array of air holes running along their entire length and confining light in the defects of the periodic structure, have extraordinary properties compared to conventional optical fibers such as endless single-mode operation, unusual chromatic dispersion, high birefringence, high or low non-linearity, *etc.* Additionally, the optical properties of silica-air PCFs are determined by the position, size, and shape of the air holes, and can be extended by filling the holes with materials such as liquid crystals [3], semiconductors [4], or metals [5,6], *etc.*, and consequently have a number of advantages for sensing applications.

Sensors based on dual-core PCFs configuration have been shown to be capable of achieving enhanced sensitivity for refractive index (RI) sensing [7–9]. The air holes in one core are selectively filled with an aqueous analyte to tune the coupling properties of the dual-core PCF. The holes in the PCF are typically small and thus only minute sample volumes are required to achieve high sensitivity. Therefore, the dual-core PCFs are very well suited for a tiny sample sensing. However, selective filling of the fiber holes is difficult and time-consuming work.

Surface plasmon resonance (SPR), characterized by its high sensitivity to variations in the refractive index of the surrounding dielectric, and has been implemented in numerous sensing structures, from the classical prism configuration [10] to waveguide based structures, from planar metallic layers to metallic coating around fibers [11]. Since it was demonstrated that high-pressure chemical deposition techniques can be used to uniformly coat the hole surfaces of a PCF with a variety of materials [12], the excitation of SPRs in PCFs with coated metal inclusions have been actively studied using both experimental [13,14] and numerical methods [15–20]. The PCF-based SPR sensors for liquid substances can be constructed by infiltrating the analyte into the metal-coated holes of the PCF. So far, numerical analyses of such SPR sensors based on PCFs have been reported [16–20]. In these proposed designs, however, selective filling of the fiber holes are required. Moreover, the structures are also difficult to coat with the metal film.

SPRs supported by metallic nanowires have been reported in recent years [5,21]. In metallic nanowire-filled PCFs, plasmonic modes can form on the metallic nanowire [6,22], and the resonance peak will be excited when the core mode couples to leaky plasmonic modes at particular frequencies. In this paper, we design a SPR temperature sensor using a commercially available six-hole PCF. All the air holes of the PCF are filled with a silver nanowire and a large thermo-optic coefficient liquid mixture (ethanol and chloroform). The filled nanowires can support resonance peaks, and the filled mixture as sensing medium can make the peak extremely sensitive to temperature. Temperature variations will induce changes of refractive indices of the mixture, thus leading to the shift of the resonance peak. By measuring the peak shift, temperature change can be detected. The refractive index of the mixture is close to that of the PCF material, which will enhance the coupling efficiency between core modes and plasmonic modes, and increase the shift of the peak for the same index change. Numerical simulation demonstrates that a temperature sensitivity as high as 4 nm/K can be achieved, which is much higher than that of fiber Bragg grating (0.01 nm/K), liquid-sealed PCF (0.17 nm/K), and SPR based selectively coated PCF (0.72 nm/K) optic temperature sensors [23–25]. Moreover, the maximal sensitivity of the sensor is relatively stable with randomly filled nanowires, which will be very convenient for the sensor fabrication and application.

## 2. Sensor Design and Numerical Modeling

**Figure 1.**
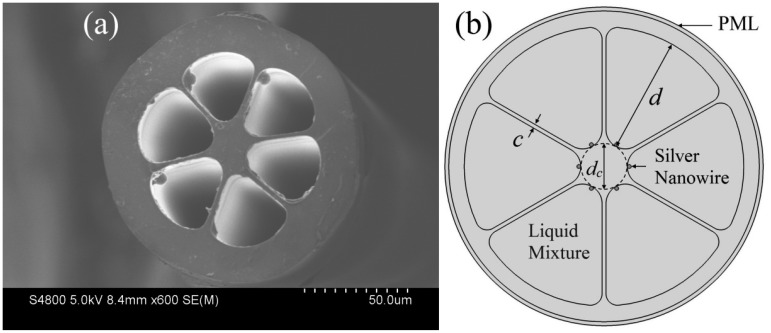
(**a**) Cross-section image of a six-hole PCF; (**b**) Schematic of the proposed sensor fiber. The silver nanowires indicated by megascopic blue dots. Parameters *c*, *d_c_* and *d* denote the thickness the core strut, the diameters of the core and the holes, respectively.

Six-hole PCFs, also termed grapefruit fibers, are shown in Figure 1a [26]. For sensor operation, each of the holes is filled with one silver nanowire of 300 nm diameter and a large thermo-optic coefficient liquid. The liquid with silver nanowires can be full-filled into the air holes by capillary force and air pressure. The fabrication should be easy because the air holes of the PCF are large enough. Then, the two ends of the liquid-filled PCF are spliced to standard single-mode fibers (SMF) using a commercial fusion splicer [24,27]. The cross section of the resulting structure is schematically depicted in Figure 1b. When the phase matching is satisfied at a certain wavelength regime, the energy of a core mode is transferred to a plasmonic mode. As the plasmonic mode is highly lossy, a significant increase in loss will be observed at this wavelength regime.

**Figure 2.**
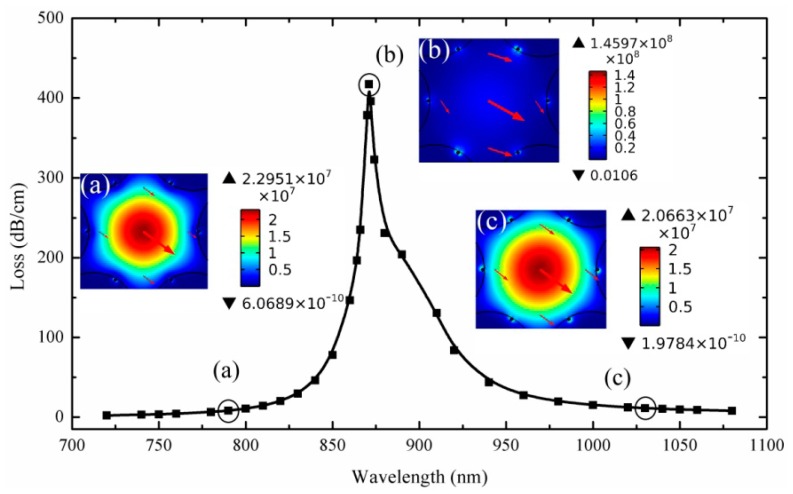
Calculated loss spectra of the core modes with the refractive index of the liquid at 1.4. Insets show the electric field (E field) distributions of the core modes, and the arrows indicate the polarized direction of electric field.

As shown in Figure 1b, the thickness of the core struts is *c* = 1 μm. The diameters of the core and the holes are *d_c_* = 12 μm and *d* = 30 μm, respectively. The refractive index of the PCF material is assumed to be 1.45 (fused silica), and the refractive index of the silver is given by the Handbook of Optics [28]. The optical fiber transmission loss (*α_loss_*) is proportional to the imaginary part of the effective index (*n_eff_*) according to the relation [21]:

(1) αloss=10lge·2k0Im[neff]=8.686·k0Im[neff](dB/m)

here, *k*_0_ = 2π/*λ* is the wavenumber with *λ* being the free-space wavelength. The electromagnetic mode (including the imaginary part of the effective index) of the sensor fiber is solved with the finite element method (FEM) by using COMSOL Multiphysics software. For the FEM modeling, we use the perfectly matched layer (PML) to matching the outmost layer (see Figure 1b), and the triangular sub-domain to discretize the computation area. Figure 2 shows the calculated loss spectra of the core modes in the wavelength range of 700–1100 nm when the refractive index of the liquid is 1.4. The points of the spectra are obtained from the Equation (1). Here, we use the Gaussian-like modes as the core modes [15,17], and it is best suited for the excitation by standard Gaussian laser sources. As shown in Figure 2, the resonance peak located at 871 nm defined by increase in the core mode propagation losses. The losses of a core mode increasing dramatically due to the energy transfer into the lossy plasmonic mode. The electric field (E field) distributions of the core modes (insets in Figure 2) show clearly the energy transferred between the two modes. At non-resonance wavelengths, the core modes (inset (a) and inset (c)) are fundamental mode (HE_11_), and the energy is mainly confined in the core area. At resonance wavelengths, the core modes and the plasmonic modes become strongly mixed (inset (b)), and the energy transfer into the plasmonic modes. Thus, an obvious peak of the core mode loss spectrum is observed at this wavelength regime.

## 3. Results and Discussion

### 3.1. RI Sensitivities of Sensors

**Figure 3.**
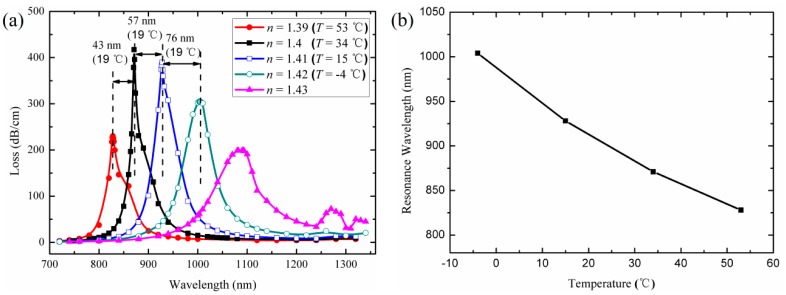
(**a**) Calculated loss spectra of the core modes with different refractive indices of the liquid (temperatures); (**b**) Resonance wavelength curves of the sensor when the volume ratio of the ethanol and chloroform is 4:6.

To investigate the RI sensitivity of the sensor, we present loss spectra of the core modes in the wavelength range of 700–1400 nm for the different refractive indices of the liquid in Figure 3a. The shift of the resonance peak is from 828 nm for *n* = 1.39 to 871 nm for *n* = 1.4 and 928 nm for *n* = 1.41. The increasing shifts in resonance wavelength for the same index change suggest higher sensitivity for the detection range for high *n*_liquid_ than for that for low *n*_liquid_. According to the numerical calculations, the maximal sensitivity is 7600 nm/Refractive Index Unit (RIU) for *n* = 1.41–1.42 detection range. Theoretically, phase matching (resonance) constitutes equating the effective refractive indices of the core mode and plasmonic mode at a given wavelength of operation. The effective refractive index of a core mode is close to that of a core material, (*n* = 1.45 for silica) and the effective refractive index of a plasmonic mode is close to that of a bordering liquid [16,17]. Therefore, the high refractive index of the liquid will enhance the coupling efficiency between the two modes and the sensitivity. However, it is important to note that only one primary peak is observed for the sensor when the refractive index of the liquid is smaller than 1.42. When the index value increases, secondary peaks will appear at long wavelengths. The secondary peaks may introduce noise and make the detection of high *n*_liquid_ more difficult. Moreover, the increasing index of the liquid will result in the low refractive index-contrast of the PCF, which lead to higher losses of the core modes. Therefore, to achieve the high sensitivity, the refractive index of the filled liquid (the sensing medium) should not exceed 1.42.

### 3.2. Temperature Sensitivities of Sensors

To enhance the sensitivity for temperature sensing applications, a dielectric material with a high-value thermo-optic coefficient (*dn*/*dT*) is needed in the PCF holes. Here, we assume a liquid mixture of ethanol and chloroform is filled into the fiber holes as sensing medium (see Figure 1b). The ethanol introduced is to lower the refractive index of the sensing medium, because the chloroform has a large refractive index (*n* = 1.44). The refractive index of the liquid is evaluated by [29]:

(2) $$n=n_{0}+dn/dT\cdot (T-T_{0})$$

here *n*_0_ is the refractive index of the liquid at the reference temperature *T*_0_. We neglect the material dispersion of the liquid and assume *n*_0_ are 1.36 for ethanol and 1.44 for chloroform for the spectral regime from 700 nm to 1400 nm at 20 °C. The thermo-optical coefficients *dn*/*dT* amount to −3.94 × 10^−4^/K for ethanol [30] and −6.328 × 10^−4^/K for chloroform [31], respectively. Furthermore, they are assumed independent with the incident wavelength and temperature. In contrast to the value of the liquids, the thermo-optical coefficient of the fused silica (∼10^−6^/K) [26] and the silver are lower than the liquids and are therefore not taken into consideration. The Lorentz-Lorenz equation is used for the refractive index of the liquid mixture [32]:

(3) $$\frac{n^{2}-1}{n^{2}+2}=\phi_{1}\frac{n_{1}^{2}-1}{n_{1}^{2}+2}+\phi_{2}\frac{n_{2}^{2}-1}{n_{2}^{2}+2}$$

here, *n*, *n*_1_ and *n*_2_ are the refractive index of the solution and the constituents, respectively. *φ*_1_ and *φ*_2_ are the volume fractions of the constituents and *φ*_2_ can be replaced by 1 − *φ*_1_.

The mixture of ethanol and chloroform with a volume ratio of 4:6 is assumed to be filled into the fiber holes. Figure 3 shows the loss spectra of core modes and the resonance wavelength curve of the sensor with the mixture at 53 °C, 34 °C, 15 °C, and −4 °C (the mixture still in liquid-phase) temperatures. In this case, sensitivity is defined as [25]:

(4) $$S_{\lambda }[\text{nm}/K]=\Delta \lambda_{peak}/\Delta T$$

The maximal sensitivity is 4 nm/K for *T* = −4–15 °C detection range. Besides, the most sensitive range of the sensor can be tuned to a desired value by changing the volume ratios of the constituents in the mixture. For example, if the maximal sensitivity is expected at *T* = 20 °C, according to the Equations (2) and (3), the volume ratio of the ethanol and chloroform should be taken 3:7 (*n* = 1.42 at 20 °C).

### 3.3. Temperature Sensitivities of PCFs Randomly Filled with Silver Nanowires

In consideration of the actual operation, it is difficult to regularly fill PCFs with silver nanowires like the structure shown in Figure 1b. Mostly, the nanowires are filled randomly and irregularly. To investigate the effect of the randomness of filled nanowires on the sensitivity of the sensor, firstly, we consider the influence of the nanowire positions on the resonance peak.

#### 3.3.1. Influence of Silver Nanowire Positions on Resonance Peaks

**Figure 4.**
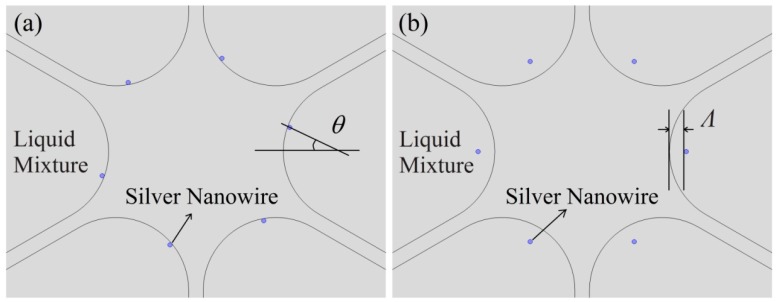
(**a**) The silver nanowires are placed on the surfaces of the fiber holes and rotated *θ* degrees; (**b**) The silver nanowires leave the surfaces of the fiber holes, and the relative distance between the surfaces is *Λ*. The silver nanowires indicated by blue dots.

**Figure 5.**
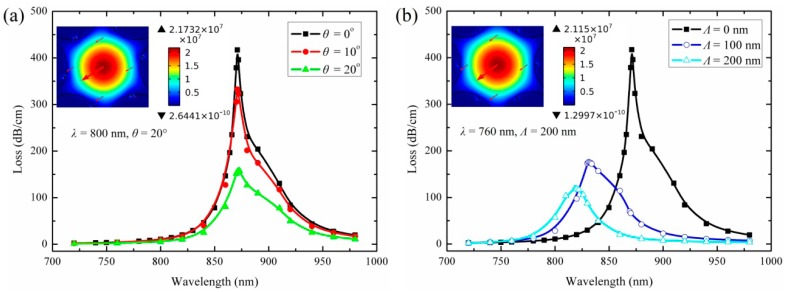
(**a**) Loss spectra for the structure shown in Figure 4a with *n*_liquid_ = 1.4 at different angles *θ*. The inset shows the electric field (E field) distribution of the core mode with *θ* = 20° at 800 nm; (**b**) Loss spectra for the structure shown in Figure 4b with *n*_liquid_ = 1.4 at different distances *Λ*. The inset shows the electric field (E field) distribution of the core mode with *Λ* = 200 nm at 760 nm. The arrows indicate the direction of polarization of the electric field.

Figure 4a,b shows the schematics of the PCF filled with silver nanowires at different positions (indicated by blue dots). And the simulation results for the loss spectra of the structures with liquid index at 1.4 are plotted in Figure 5.

As shown in Figure 5a, the resonance wavelength is not changed when the surface nanowires deviate from the geometrical center of the fiber holes. However, the resonance intensity decreases as the angle *θ* increases. In Figure 5b, the wavelength and intensity of the resonance peak are all changed when the nanowires leave from the surfaces of the fiber holes. The peak shifts to a shorter wavelength and the intensity decreases, as the distance *Λ* increases. In short, the resonance wavelength will not be changed as long as the nanowires are still on the surfaces of the holes. The result will be valuable for design and fabrication of such sensors.

#### 3.3.2. Influence of Random of Filled Silver Nanowires on Sensitivities

**Figure 6.**
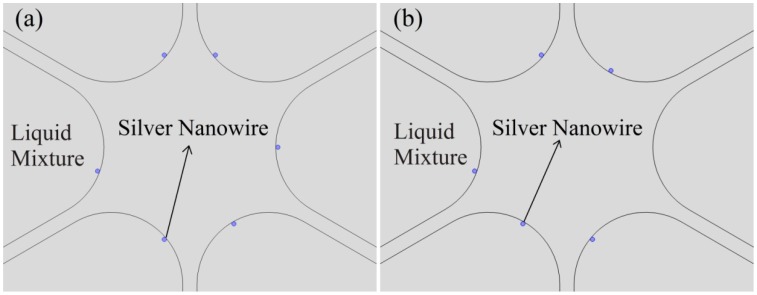
(**a**) Each of the fiber holes is randomly filled with one silver nanowire; (**b**) One of the holes is missed during the filling. The silver nanowires are indicated by blue dots.

We now investigate the sensitivity of sensors based on PCFs randomly filled with the nanowires. According to the results of the Figure 5, we propose the PCFs randomly filled with the nanowires like the structures shown in Figure 6. All the silver nanowires are randomly placed on the surfaces of the fiber holes. Besides, it is unlikely that the nanowires suspended in the liquid (leave from the holes surface) because of the gravity effect. As shown in Figure 6a, each of the fiber holes is filled with nanowires at random positions. And in Figure 6b, one of the PCF holes is missed to fill. These can be more close to the condition of that the holes filled with nanowires during the actual operation.

**Figure 7.**
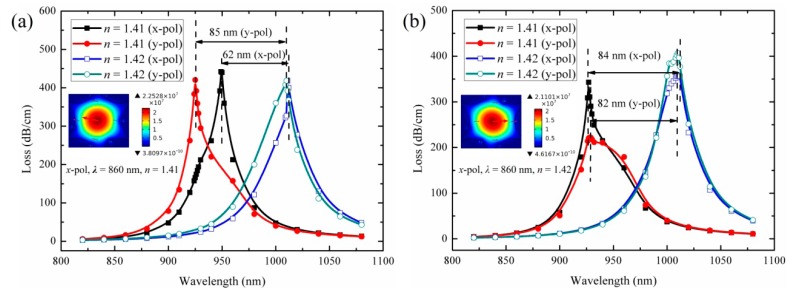
(**a**) Loss spectra for the structure shown in Figure 6a with liquid index at 1.41 and 1.42. The inset shows the electric field (E field) distribution of the *x*-polarized core mode with *n*_liquid_ = 1.41 at 860 nm; (**b**) Loss spectra for the structure shown in Figure 6b with liquid index at 1.41 and 1.42. The inset shows the electric field (E field) distribution of the *x*-polarized core mode with *n*_liquid_ = 1.42 at 860 nm. The arrows indicate the direction of polarization of the electric field.

Figure 7 shows the loss spectra of the *x*-polarized and *y*-polarized core modes for the structures shown in Figure 6 when the refractive index of the liquid are 1.41 and 1.42 (corresponding to the temperatures are 15 °C and −4 °C for the 4:6 volume ratio of the filled mixture). Unlike the regular filled silver nanowires that support a single resonance peak overlapped with *x*-polarized and *y*-polarized core modes, the random filled silver nanowires can support two separate peaks which are *x*-polarized and *y*-polarized core modes. As shown in Figure 7, the maximal sensitivity in Figure 7a is very close to that in Figure 7b, which is 8500 nm/RIU (4.47 nm/K) of *y*-polarized core modes in Figure 7a and 8400 nm/RIU (4.42 nm/K) of *x*-polarized core modes in Figure 7b. The maximal sensitivity in Figure 7 are different from that in Figure 6 due to the random arrangements of the filled nanowires, which split one resonance peak into two peaks (*x*-polarized and *y*-polarized resonance peaks) and cause the changing of the resonance wavelength and the sensitivity. Nevertheless, the maximal sensitivity of the sensor is relatively stable even with the randomly filled nanowires. This will be very convenient for the practical implementation of the experiment.

## 4. Conclusions

We have proposed a SPR temperature sensor design based on filling a six-hole PCF with silver nanowires and a large thermo-optic coefficient liquid mixture (ethanol and chloroform). The filled silver nanowires can also support resonance peaks just like the coated metal ones can. Temperature variations will change the refractive index of the filled mixture and influence the coupling efficiencies between core modes and plasmonic modes, thus leading to a peak shift. By measuring the peak shift, temperature changes can be detected. A temperature sensitivity as high as 4 nm/K has been demonstrated in the proposed structure, which is much higher than many formerly demonstrated structures. The presented design has the following advantages: firstly, the PCF filled with the metallic nanowires should be easier to implement than that coated with a metal film. The maximal sensitivity of the sensor is relatively stable with the randomly filled nanowires, which will be very convenient for the sensor fabrication and application. Secondly, the refractive index of the liquid mixture is close to that of the PCF material, which will enhance the coupling efficiency between the core mode and the plasmonic mode and increase the sensitivity. Finally, by adjusting the volume ratios of the constituents in the mixture, the most sensitivity range of the sensor can be tuned to a desired value.
